# Event-by-event cumulants of azimuthal angles

**DOI:** 10.1140/epjc/s10052-022-10740-z

**Published:** 2022-09-05

**Authors:** Ante Bilandzic

**Affiliations:** Physik Department, Technische Universität München, Munich, Germany

## Abstract

We further develop the recently proposed event-by-event cumulants of azimuthal angles. The role of reflection symmetry, permutation symmetry, frame independence, and relabeling of particle indices in the cumulant expansion is discussed in detail. We argue that mathematical and statistical properties of cumulants are preserved if cumulants of azimuthal angles are defined event-by-event in terms of single-event averages of azimuthal angles, while they are violated in the traditional approach in which cumulants are defined in terms of all-event averages. We derive for the first time the example analytic solutions for the contribution of combinatorial background in the measured two- and three-particle correlations. We demonstrate that these solutions for the combinatorial background are universal, as they can be written generically in terms of multiplicity-dependent combinatorial weights and marginal probability density functions of starting multivariate distribution. The new general results between multiparticle azimuthal correlators and flow amplitudes and symmetry planes are presented.

## Introduction

In ultrarelativistic heavy-ion collisions, the properties of an extreme state of nuclear matter, dubbed quark–gluon plasma, can be studied in a controlled environment [1–3]. Among various experimental observables used to probe its properties, in the present work we focus exclusively on multivariate cumulants [4]. They have been regularly applied at both the Relativistic Heavy Ion Collider (RHIC) and Large Hadron Collider (LHC) experiments for the measurement of anisotropic flow phenomena [5, 6] and in femtoscopic studies [7, 8]. In the context of flow analyses, the cumulant expansion has been performed traditionally on azimuthal angles [9–12], and only recently also directly on flow amplitudes [13–15]. Both the statistical and systematic uncertainties of these measurements are suppressed with inverse powers of multiplicity, which means that multivariate cumulants are a precision tool only in heavy-ion datasets characterized by large values of multiplicity. On the other hand, their measurements in the collisions of smaller objects, such as proton–proton collisions, are unreliable, and there is still no consensus in the field on how to interpret them in this environment.

The mathematical properties of cumulants are rigorous and well established (for a recent concise review and summary, see Ref. [15]). The non-trivial physics information which can be extracted from cumulants stems from the observation that a cumulant is identically zero if one of the variables in it is statistically independent of the others [4]. However, this statement is not a mathematical equivalence, and therefore Theorem 1 from Ref. [4] cannot be used to conclude that variables in the cumulant definition are statistically independent of each other if the cumulant is zero. On the contrary, a cumulant is not zero if and only if all variables in it are statistically connected [4], i.e. there exists a genuine multivariate correlation among all variables in the cumulant which cannot be written as a superposition of correlations among a smaller number of variables. By definition, each higher-order cumulant extracts a piece of new and independent information that is not accessible at lower orders.

The traditional cumulant expansion over azimuthal angles yields the final expressions that contain only the isotropic all-event averages of azimuthal correlators [9, 10]. All non-isotropic averages are identically zero due to random fluctuations of the impact parameter vector for detectors which have uniform azimuthal acceptance [9, 10]. However, it was recently realized that these results for cumulants of azimuthal angles in which the non-isotropic azimuthal correlators are missing no longer satisfy the fundamental properties of cumulants [13, 15]. The reason for this failure is that mathematical and statistical properties of cumulants are in general preserved only if all terms in the cumulant expansion are kept. As soon as there are underlying symmetries due to which some terms in the cumulant expansion are lost, their mathematical and statistical properties are invalidated, while their physical interpretation can be completely different.

An alternative definition of cumulants of azimuthal angles was recently proposed in Ref. [15]. Instead of defining cumulants in terms of all-event averages of azimuthal angles, cumulants are defined in terms of single-event averages. These new *event-by-event cumulants of azimuthal angles* satisfy all mathematical and statistical properties of cumulants. However, their direct application and measurement are plagued by contributions from the combinatorial background, which are very difficult to remove completely. The standard and approximate mixed-event technique used in high-energy physics to remove a combinatorial background is not applicable for azimuthal angles.

The main motivation for the current draft is an attempt to make further progress with these recently introduced event-by-event cumulants of azimuthal angles. As our main new result, we show how the contributions from the combinatorial background in the measured two- and three-particle correlations can be solved analytically by using universal combinatorial weights which depend only on multiplicity and using marginal probability density functions (p.d.f.). These example solutions can be straightforwardly generalized to correlations involving more than three particles and for cases when multiple particles were misidentified in the calculated correlators. The generalization will be presented in our future work.

The rest of the paper is organized as follows: In Sect. 2, we discuss the role of various underlying symmetries which can appear in cumulant expansion. In Sect. 3, we discuss how the presence of a combinatorial background affects measurements obtained with correlation techniques. The new set of general results between multiparticle azimuthal correlators and flow amplitudes and symmetry planes are presented in Appendix A.

## Role of symmetries

In this section we discuss the role of a few symmetries which can appear in the cumulant expansion. As our main conclusion, we demonstrate that genuine multivariate correlations can be reliably extracted with cumulants only if there are no underlying symmetries due to which some terms in the cumulant expansion are identically zero. This topic is of great relevance particularly for anisotropic flow analyses in high-energy physics, where most of the cumulants used now suffer from this problem. In what follows next, we will frequently use the phrase “p.d.f. does not factorize,” with the following meaning: The starting *n*-variate (or joint) p.d.f. $f(x_1,x_2,\ldots ,x_n)$ cannot be decomposed into the product of marginal p.d.f.’s corresponding to some set partition of $x_1,x_2,\ldots ,x_n$. For instance, a factorizable three-variate p.d.f. would permit the decompositions $f(x_1,x_2,x_3) = f_{{x_1}{x_2}}(x_1,x_2)f_{x_3}(x_3)$, or $f(x_1,x_2,x_3) = f_{x_1}(x_1)f_{x_2}(x_2)f_{x_3}(x_3)$, etc., where $f_{x_{i}x_{j}}$ and $f_{x_i}$ are two- and single-variate marginal p.d.f.’s of the starting three-variate p.d.f. $f(x_1,x_2,x_3)$. We use indices for p.d.f.’s to indicate that their functional forms can be different, instead of naming them differently. For ease of notation, we drop indices only when we refer to the starting *n*-variate p.d.f. of all $x_1,x_2,\ldots ,x_n$ variables in question. Theorem 1 from Ref. [4] states that the *n*-variate cumulant is identically zero if the underlying *n*-variate p.d.f. is factorizable. Physically, this means that there are no genuine correlations among all *n* particles in the system, i.e. *n*-particle correlation is just a trivial superposition of a correlation involving less than *n* particles. Mathematically, it is possible to find a set partition of $x_1,x_2,\ldots ,x_n$ in which subsets are statistically independent of each other. We now demonstrate that, in general, the opposite is not true, i.e. the cumulant can be identically zero because of underlying symmetries and not because variables on which the cumulant expansion has been performed are statistically independent.

### Reflection symmetry

We start the discussion with the following well-known argument. If $f(x_1,x_2), x_1,x_2\in (-\infty ,\infty ),$ is a two-variate p.d.f. which does not factorize, the corresponding two-variate cumulant is not zero. Based on this observation, we conclude that the two variables $x_1$ and $x_2$ are not statistically independent, i.e. there exists a genuine two-body correlation between them. This conclusion fails, however, if in addition the starting p.d.f. $f(x_1,x_2)$ has the following reflection symmetry: $f(x_1,x_2) = f(-x_1,x_2)$. Because of this symmetry, the corresponding two-variate cumulant, $\langle x_1 x_2\rangle - \langle x_1\rangle \langle x_2\rangle$, is now identically zero, simply because both $\langle x_1\rangle \equiv \int \!\!\int x_1\,f(x_{1},x_{2})\,\mathrm{{d}}x_{1}\mathrm{{d}}x_{2}$ and $\langle x_1x_2\rangle \equiv \int \!\!\int x_1x_2\,f(x_{1},x_{2})\,\mathrm{{d}}x_{1}\mathrm{{d}}x_{2}$ are identically zero. But this result does not imply that $x_1$ and $x_2$ are statistically independent—the starting p.d.f. is still not factorizable. This simple example clearly illustrates the failure of cumulants in the presence of underlying symmetries [16].

We now generalize this argument to higher orders. If $f(x_1,x_2,\ldots ,x_n), x_1,x_2,\ldots ,x_n\in (-\infty ,\infty ),$ is an *n*-variate p.d.f. which does not factorize, and if we again assume that there is a reflection symmetry in one variable $f(x_1,x_2,\ldots ) = f(-x_1,x_2,\ldots )$, it still follows that identically $\langle x_1\rangle = 0$, since

1 $$\begin{aligned} \langle x_1\rangle\equiv & {} \int _{-\infty }^{\infty }\cdots \int _{0}^{\infty }x_1f(x_1,x_2,\ldots ,x_n)\,\mathrm{{d}}x_1\cdots \mathrm{{d}}x_n\nonumber \\= & {} \int _{-\infty }^{0}\cdots \int _{0}^{\infty }x_1f(x_1,x_2,\ldots ,x_n)\,\mathrm{{d}}x_1\cdots \mathrm{{d}}x_n \nonumber \\&\quad + \int _{0}^{\infty }\cdots \int _{0}^{\infty }x_1f(x_1,x_2,\ldots ,x_n)\,\mathrm{{d}}x_1\cdots \mathrm{{d}}x_n\nonumber \\= & {} \int _{\infty }^{0}\cdots \int _{0}^{\infty }(-x_1)f(-x_1,x_2,\ldots ,x_n)\,(-\mathrm{{d}}x_1)\cdots \mathrm{{d}}x_n \nonumber \\&\quad + \int _{0}^{\infty }\cdots \int _{0}^{\infty }x_1f(x_1,x_2,\ldots ,x_n)\,\mathrm{{d}}x_1\cdots \mathrm{{d}}x_n\nonumber \\= & {} -\int _{0}^{\infty }\cdots \int _{0}^{\infty }x_1f(-x_1,x_2,\ldots ,x_n)\,\mathrm{{d}}x_1\cdots \mathrm{{d}}x_n \nonumber \\&\quad + \int _{0}^{\infty }\cdots \int _{0}^{\infty }x_1f(x_1,x_2,\ldots ,x_n)\,\mathrm{{d}}x_1\cdots \mathrm{{d}}x_n\nonumber \\= & {} -\int _{0}^{\infty }\cdots \int _{0}^{\infty }x_1f(x_1,x_2,\ldots ,x_n)\,\mathrm{{d}}x_1\cdots \mathrm{{d}}x_n \nonumber \\&\quad + \int _{0}^{\infty }\cdots \int _{0}^{\infty }x_1f(x_1,x_2,\ldots ,x_n)\,\mathrm{{d}}x_1\cdots \mathrm{{d}}x_n\nonumber \\= & {} 0\,. \end{aligned}$$

The above derivation holds true if we add some of the remaining variables $x_j, x_k, \ldots$ into the correlator, i.e. we have

2 $$\begin{aligned} \langle x_1x_jx_k\cdots \rangle = 0\,,\quad \forall j,k,\ldots \ne 1\,. \end{aligned}$$

Each term in the cumulant expansion is a possible partition of the set $x_1,x_2,\ldots ,x_n$, in which in addition the averages have been taken over each subset. Therefore, in each term in the cumulant expansion there is always exactly one average which involves $x_1$, which is identically zero if the underlying *n*-variate p.d.f. has a reflection symmetry $f(x_1,x_2,\ldots ) = f(-x_1,x_2,\ldots )$ in that variable. From this we can conclude that due to reflection symmetry in any of the variables, cumulants at any order can be trivial and identical to zero. This does not imply that variables $x_1,x_2,\ldots ,x_n$ are statistically independent; instead, this is an extreme example in which the cumulant expansion is invalidated at all orders due to underlying symmetries.

To resolve between the two possibilities, the following additional cross-check needs to be performed in practice: If the underlying *n*-variate p.d.f. factorizes, cumulants are identically zero, irrespective of the choice of sample space for *n* variables $x_1,x_2,\ldots ,x_n$. In this case, cumulants will be identically zero for any choice of boundaries in $x_1\in (x_{1,\mathrm min},x_{1,\mathrm max})$, $x_2\in (x_{2,\mathrm min},x_{2,\mathrm max})$, etc., and it can be safely concluded that there are no genuine multivariate correlations among all *n* variables. On the other hand, if cumulants are identically zero due to reflection symmetry, that will occur only for some specific choice of boundaries in $x_1\in (x_{1,\mathrm min},x_{1,\mathrm max})$, $x_2\in (x_{2,\mathrm min},x_{2,\mathrm max})$, etc.

### Permutation symmetry

Next, we consider the role of permutation symmetry in the starting *n*-variate p.d.f. $f(x_1,x_2,\ldots ,x_n)$. For the sake of clarity, we present the argument for the simplest two-variate case, since the generalization to higher orders is trivial.

If for the starting two-variate p.d.f. we have that $f(x,y) = f(y,x)$, and if the sampling spaces of *x* and *y* are identical, it follows immediately that marginal p.d.f.’s are the same, since

3 $$\begin{aligned} f_x(x)\equiv & {} \int _y f(x,y)\,\mathrm{{d}}y\nonumber \\= & {} \int _x f(y,x)\,\mathrm{{d}}x\quad \mathrm{(trivial\ relabeling}\ y\rightarrow x)\nonumber \\= & {} \int _x f(x,y)\,\mathrm{{d}}x\quad \mathrm{(permutation\ symmetry)}\nonumber \\= & {} f_y(y)\,. \end{aligned}$$

This conclusion is used frequently later in the paper.

### Frame independence

A mandatory requirement which any physics observable must fulfill is its independence from the rotations of a coordinate system in the laboratory frame. We can inspect the role of rotations already at the level of two-particle cumulants of azimuthal angles. In the traditional approach, two-particle cumulants of azimuthal angles are defined as [9, 10]:

4 $$\begin{aligned} c_n\{2\}\equiv & {} \langle \langle e^{in(\varphi _1\!-\!\varphi _2)}\rangle \rangle -\langle \langle e^{in\varphi _1}\rangle \rangle \langle \langle e^{-in\varphi _2}\rangle \rangle \,. \end{aligned}$$

The double angular brackets indicate that in the first step, averaging is performed over all distinct combinations of two azimuthal angles within the same event, and then these results are averaged over all events. If we now rotate randomly by angle $\alpha$ from one event to another the coordinate system in which azimuthal angles are measured, then both $\langle \langle e^{in\varphi _1}\rangle \rangle$ and $\langle \langle e^{-in\varphi _2}\rangle \rangle$ are trivially averaged to zero [9, 10]. The reason is the following mathematical identity:

5 $$\begin{aligned} \langle \langle e^{in(\varphi _1+\alpha )}\rangle \rangle = \langle \langle e^{in\alpha }\rangle \rangle \langle \langle e^{in\varphi _1}\rangle \rangle = 0 \times \langle \langle e^{in\varphi _1}\rangle \rangle = 0\,. \end{aligned}$$

In practice, the above identity is a direct consequence of random event-by-event fluctuations of the impact parameter vector. Only the first term in Eq. (4) is invariant with respect to the rotations. In this traditional approach, the rotational invariance of cumulants was achieved by simply averaging out all non-isotropic terms. However, by following such a procedure, especially at higher orders, most of the terms in the cumulant expansion are lost, and the final expressions are no longer valid cumulants of azimuthal angles.

If we change the definition and instead define cumulants of azimuthal angles event-by-event [15]

6 $$\begin{aligned} \kappa _{11}\equiv & {} \langle e^{in(\varphi _1\!-\!\varphi _2)}\rangle -\langle e^{in\varphi _1}\rangle \langle e^{-in\varphi _2}\rangle \,, \end{aligned}$$

the rotational invariance is satisfied by definition, and all terms in the cumulant expansion are kept. We now have that

7 $$\begin{aligned} \langle e^{in(\varphi _1+\alpha )}\rangle \langle e^{-in(\varphi _2+\alpha )}\rangle= & {} e^{in(\alpha - \alpha )}\langle e^{in\varphi _1}\rangle \langle e^{-in\varphi _2}\rangle \nonumber \\ {}= & {} \langle e^{in\varphi _1}\rangle \langle e^{-in\varphi _2}\rangle \,. \end{aligned}$$

This argument generalizes trivially to higher orders. As our new general result, we argue that all terms in the cumulant expansion can be kept, and simultaneously the rotational invariance of cumulants can be satisfied, only if cumulants of azimuthal angles are defined event-by-event in terms of single-event averages. This conceptual difference in the definition of cumulants of azimuthal angles (all-event averages vs. single-event averages) yields different final results and therefore has non-trivial consequences.

### Relabeling

Another common practice in the field is to group together in the final expressions for cumulants all azimuthal correlators which are identical, apart from relabeling of indices of azimuthal angles. For instance, in the derivation of the traditional expression for a four-particle cumulant, there is the following intermediate result [9, 10]

8 $$\begin{aligned} c_n\{4\}= & {} \langle \langle e^ {in(\varphi _1\!+\!\varphi _2\!-\!\varphi _3-\!\varphi _4)}\rangle \rangle \nonumber \\&-\langle \langle e^{ in(\varphi _1\!-\!\varphi _3)}\rangle \rangle \langle \langle e^{in(\varphi _2\!-\!\varphi _4)}\rangle \rangle \nonumber \\&-\langle \langle e^{in(\varphi _1\!-\!\varphi _4)}\rangle \rangle \langle \langle e^{in(\varphi _2\!-\!\varphi _3)}\rangle \rangle \,. \end{aligned}$$

All two-particle correlators in the above equation are estimated from the same sample of azimuthal angles. Therefore, they are mathematically identical, and to reflect that, they are grouped together to obtain the final expression

9 $$\begin{aligned} c_n\{4\}= & {} \langle \langle e^{in(\varphi _1\!+\!\varphi _2\!-\!\varphi _3-\!\varphi _4)}\rangle \rangle -2\langle \langle e^{in(\varphi _1\!-\!\varphi _2)}\rangle \rangle ^2\,. \end{aligned}$$

We now argue that such a practice conflicts with the strict mathematical properties of cumulants. In general, the *n*-variate cumulant $\kappa _{\nu _1,\ldots ,\nu _n}$ is by definition a sum in which each individual summand can be thought of as one possible partition of the set consisting of *n* starting variables $\{x_1,...,x_n\}$ [4]. After relabeling variable indices in the final expressions for cumulants, this fundamental property of cumulants is lost.

This problem is closely related to the general problem of combinatorial background, which we tackle in the next section. Relabeling discussed here amounts essentially to ignoring all the non-trivial effects of the combinatorial background. The formalism of cumulants can be easily applied only in cases when combinatorial background plays no role (e.g. in the study of event-by-event fluctuations of different flow magnitudes). However, in cases when the combinatorial background is not under control, the usage of cumulants is not that straightforward.

## Combinatorial background

In this section, we investigate the role of combinatorial background in analyses that rely on the use of correlation techniques and cumulants. We keep the discussion general, and therefore our main conclusions are applicable to diverse fields of interest (anisotropic flow, femtoscopy, etc.).

### Trivial multiplicity scaling

One of the well-known results in high-energy physics is that in the absence of collective phenomena, the two-particle correlators exhibit trivial multiplicity dependence, the so-called $\sim 1/M$ scaling. In fact, the breakdown of this scaling is typically proposed as a very robust signature of the collective behavior of nuclear matter that is produced.

We now decipher this trivial multiplicity scaling and demonstrate that it does not originate solely from few-particle correlations, but rather from the interplay between combinatorial background and few-particle correlations. This is a subtle yet important difference. The importance of the combinatorial background has been regularly ignored in this context, but we now demonstrate that the contributions from few-particle correlations do not exhibit this trivial multiplicity scaling in the measured two-particle correlators per se—the universal nature of $\sim 1/M$ scaling for vastly different physical sources of few-particle correlations instead stems naturally from the presence of the combinatorial background.

In general, in the measurements dominated by few-particle correlations, the nonflow contribution, $\delta _k$, in the *k*-particle correlator, $\langle \langle k\rangle \rangle$, exhibits the following universal scaling as a function of multiplicity *M* [9, 10]:

10 $$\begin{aligned} \delta _{k} \sim \frac{1}{M^{k-1}}. \end{aligned}$$

The derivation of this scaling is purely probabilistic and can be derived with the following simple argument. For the sake of simplicity, we outline the argument for four-particle correlators, but it can be trivially generalized to any higher-order correlator. In an event with multiplicity *M*, we consider four particles which are correlated through direct few-particle correlations which are not of collective origin (for instance, these four particles originate from the same resonance decay). The probability of selecting precisely these four particles into the four-particle correlator, out of *M* particles available in an event, is given by

11 $$\begin{aligned} \delta _{4} \sim \frac{3}{M-1}\frac{2}{M-2}\frac{1}{M-3} \sim \frac{1}{M^3}. \end{aligned}$$

After we have taken the first particle into the four-particle correlator to be the one from the specific four-particle resonance decay, we have three particles left from the same resonance decay out of $M-1$ in total; after we have selected the next one, there are two particles left from the same resonance decay out of $M-2$ in total, etc. If we would have instead considered the five-particle resonance decays but still used four-particle correlators in the measurements, only the numerical factors in the numerator of Eq. (11) would change, and the multiplicity dependence in the denominator would remain the same. As a consequence, the universal multiplicity scaling in Eq. (11) is always present if the measured four-particle correlators are dominated by contributions from few-particle correlations, and does not depend on the details of few-particle correlations.

Only if there are collective effects which induce correlations among all produced particles is the universal multiplicity scaling in Eq. (10) broken. By following the same probabilistic argument, we have instead that the contribution of collective correlations among all *M* particles in an event, in the measured *k*-particle correlator, is

12 $$\begin{aligned} \delta _{k} \sim 1. \end{aligned}$$

We note that there is no room for ambiguity here. For instance, in the context of anisotropic flow analyses, theoretically we can have the fine-tuned possibility that a flow harmonic itself has the following multiplicity dependence:

13 $$\begin{aligned} v(M) \sim 1/\sqrt{M}\,. \end{aligned}$$

If we now sample all azimuthal angles individually from the single-variate Fourier-like p.d.f. parameterized solely with that harmonic (we use the standard techniques and notation from Ref. [12], see also Sect. 3.1.1), the two-particle azimuthal correlator yields

14 $$\begin{aligned} \langle \langle 2 \rangle \rangle = v^2 \sim \frac{1}{M}\,, \end{aligned}$$

which is the same result as a trivial multiplicity scaling in two-particle correlators in Eq. (10). The relation between any azimuthal correlator and flow harmonics can be obtained from the general procedure outlined in Appendix A.

To distinguish these two vastly different possibilities, we must look at higher orders. For a four-particle azimuthal correlator, $\langle \langle 4 \rangle \rangle$, a contribution from few-particle correlations gives the following generic scaling $\langle \langle 4 \rangle \rangle = \delta _4 \sim 1/M^3$ (see Eq. (10)), while the non-trivial dependence of flow harmonics on multiplicity in Eq. (13) gives $\langle \langle 4 \rangle \rangle \sim 1/M^2$. Therefore, we can establish the following argument: If we observe $\langle \langle 2 \rangle \rangle \sim 1/M$, we cannot conclude based solely on that measurement whether the two-particle correlator is dominated by few-particle correlations or by correlations which are of collective origin. However, at higher orders ($k>2$, *k* is even) we have the splitting between two possibilities:

1. $\langle \langle k \rangle \rangle \sim 1/M^{k-1}$ $\Rightarrow$ the *k*-particle correlator is dominated by an interplay between few-particle correlations and combinatorial background;
2. $\langle \langle k \rangle \rangle \sim 1/M^{k/2}$ $\Rightarrow$ the *k*-particle correlator is dominated by collective effects.

We have restricted the above argument to even *k*, because for odd *k* there is an additional contribution from symmetry planes (see Appendix A), which is not relevant for the discussion presented here. We support this conclusion with the following toy Monte Carlo study.

#### The 1/*M* scaling in 2-p correlators: flow or nonflow?

**Fig. 1:**
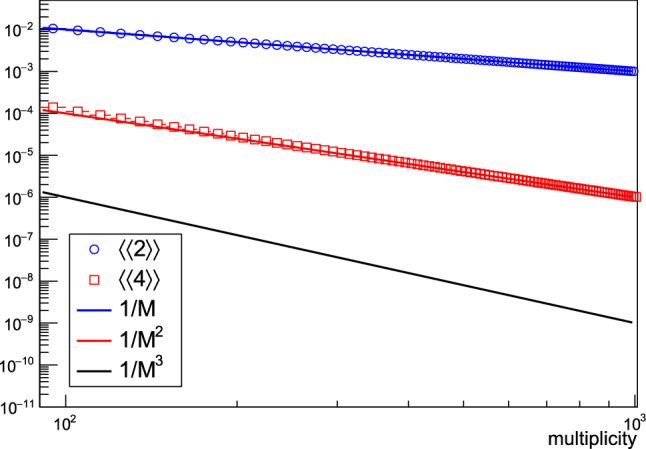
In this study, the elliptic flow $v_2$ itself exhibits $\sim 1/\sqrt{M}$ multiplicity scaling, which yields $\sim 1/M$ scaling in the two-particle correlators (blue markers), which is typically attributed to few-particle correlations. We cannot conclude based solely on the measurement of the two-particle correlator $\langle \langle 2\rangle \rangle$ whether this is a contribution from collective or non-collective effects. However, the four-particle correlator $\langle \langle 4\rangle \rangle$ (red markers) can discriminate between these two possibilities, because it follows what would be the collective flow scaling $1/M^{2}$ in this example (red line) and not a universal few-particle nonflow scaling $1/M^{3}$ (black line). To increase visibility, both horizontal and vertical axes are plotted on log scale

As the starting point in this toy Monte Carlo study, we sample the multiplicity *M* of an event. From the sampled multiplicity, we determine the elliptic flow in that event via $v_2(M) = 1/\sqrt{M}$, so that by definition in this study, $\langle \langle 2\rangle \rangle ~=~v_2^2~\sim ~1/M$, and $\langle \langle 4\rangle \rangle ~=~v_2^4~\sim ~1/M^2$. In the next step, we sample particle azimuthal angles from Fourier-like single-particle p.d.f. $f(\varphi ) = \frac{1}{2\pi }(1 + 2v_2(M)\cos (2(\varphi -\varPsi )))$, where $\varPsi$ is an event-by-event randomized symmetry plane (this is the standard procedure in the field and it resembles the random event-by-event fluctuations of impact parameter vector). Finally, from the sampled azimuthal angles we calculate the correlators $\langle \langle 2\rangle \rangle$ and $\langle \langle 4\rangle \rangle$ with the generic framework [12] as a function of multiplicity. The results of this study are presented in Fig. 1.

### Example solution for combinatorial background for two-particle correlations

While the discussion in the previous section on multiplicity scaling in the measured correlators was only qualitative and purely phenomenological, we now provide the robust mathematical treatment. In particular, we establish the mathematical procedure to obtain the analytic solution for the combinatorial background for the example process in which two particles are emitted pair-wise from the most general joint two-variate p.d.f. $f_{xy}(x,y)$. In the next section we present the solution for three-particle correlations in order to demonstrate that the same mathematical procedure is applicable at higher orders. For the sake of clarity, we consider only the most important cases of particle misidentification in the measured correlators. The general solutions which cover all possible cases of particle misidentification will be presented in our future work.

The starting problem can be formulated mathematically as follows:

1. From the general two-variate p.d.f. $f_{xy}(x,y)$ we sample particles in pairs. We perform *M*/2 samplings to obtain an event with multiplicity *M*.
2. We randomize the final sample of *M* particles.
3. What is the p.d.f. *w*(*x*, *y*) of a randomized sample if the starting p.d.f. is $f_{xy}(x,y)$?

We solve the problem for the most general case of starting p.d.f. $f_{xy}(x,y)$, i.e. we do not allow simplifications that can occur due to its factorization $f_{xy}(x,y)= f_{x}(x)f_{y}(y)$, permutation symmetry $f_{xy}(x,y) = f_{xy}(y,x)$, etc. The observables *x* and *y* are in general taken from different sample spaces and generally have different functional forms for marginal p.d.f.’s $f_{x}(x)$ and $f_{y}(y)$. To make a connection with the experiment, we can tag the starting p.d.f. $f_{xy}(x,y)$ as a “signal” and the p.d.f. *w*(*x*, *y*) after randomization as a “signal + background.” The general two-particle correlator which corresponds only to the signal is $\langle xy\rangle _{S} =\int \!\!\int \! xy\,f_{xy}(x,y)\,\mathrm{{d}}x\mathrm{{d}}y$, while the one that corresponds to both signal and background is $\langle xy\rangle _{S+B} =\int \!\!\int \! xy\,w(x,y)\,\mathrm{{d}}x\mathrm{{d}}y$.

We now demonstrate that the relation between $f_{xy}(x,y)$ and *w*(*x*, *y*) is universal, in the sense that whatever the starting functional form of $f_{xy}(x,y)$ is, the same generic equation determines the functional form of *w*(*x*, *y*). That equation involves only combinatorial weights that depend only on multiplicity *M* and marginal p.d.f.’s of $f_{xy}(x,y)$. For instance, the marginal p.d.f. $f_{x}(x)$ is the probability of obtaining *x* whatever value of *y*, and it can be obtained from the following definition $f_{x}(x)\equiv \int \!f_{xy}(x,y)\,\mathrm{{d}}y$. Further discussion about fundamental properties of marginal p.d.f.’s can be found in Ref. [16].

When particles are sampled pair-wise, and when only the most important case of particle misidentification is considered, without loss of generality we can divide the final dataset of *M* particles after randomization into the following four disjoint subsets:

1. **Diagonal:** Both particles originate from the same sampling. Only these two particles can be physically correlated, i.e. this is the signal and is therefore described with the starting p.d.f. $f_{xy}(x,y)$. Its combinatorial weight is $p_A = \frac{2!\frac{M}{2}}{M(M-1)}$.
2. **Off-diagonal:** Two particles are from different samplings and their types were identified correctly. These two particles are statistically independent, and therefore their statistical properties are described by the product of two marginal p.d.f.’s. $f_{x}(x)f_{y}(y)$. The combinatorial weight of this contribution is $p_B = 2\frac{\frac{M^2}{4}-\frac{M}{2}}{M(M-1)}$.
3. **Misidentified:** To account for the fact that identical particles are indistinguishable, we must allow the possibility that the particle which was originally sampled as *y* was reconstructed as *x*, and vice versa. For instance, if two pions are emitted from two different processes, after randomization we do not even know in principle which pion originated from which process. This case is governed by two possibilities: $f_{x}(x)f_x(y)$ and $f_{y}(y)f_y(x)$. In both cases, the combinatorial weight is the same and it reads $p_C = \frac{\frac{M^2}{4}-\frac{M}{2}}{M(M-1)}$ .

In the above expressions, $f_x$ and $f_y$ are marginal p.d.f.’s of *x* and *y*, respectively. In total, there are four disjoint subsets, and as a cross-check we can immediately see that for their respective combinatorial weights it follows that

15 $$\begin{aligned} \sum _i^4 p_i= & {} p_A + p_B + 2p_C \nonumber \\ {}= & {} \frac{2!\frac{M}{2} + 2\left( \frac{M^2}{4}-\frac{M}{2}\right) + 2\left( \frac{M^2}{4}-\frac{M}{2}\right) }{M(M-1)} = 1\,. \end{aligned}$$

We can now write the example solution for the p.d.f. of a randomized sample

16 $$\begin{aligned} w(x,y)= & {} p_A f_{xy}(x,y) + p_B f_{x}(x)f_{y}(y) \nonumber \\&\quad + p_C\big [ f_{x}(x)f_{x}(y) + f_{y}(x)f_{y}(y)\big ]\,. \end{aligned}$$

**Fig. 2:**
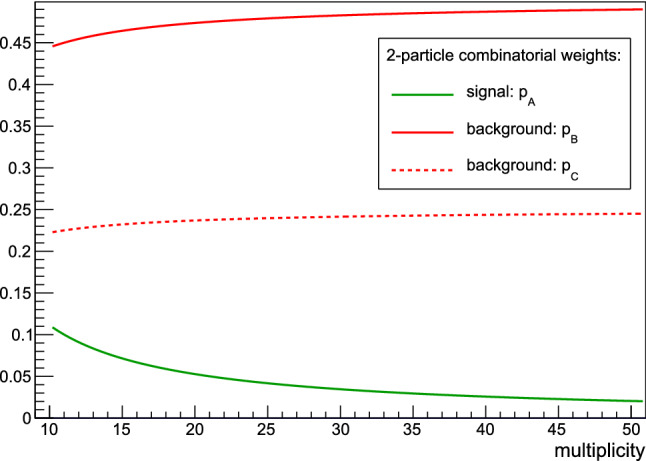
Multiplicity dependence of three distinct combinatorial weights, $p_A$, $p_{B}$ and $p_{C}$ (see the main text for their definitions and explanation), which always appear in two-particle correlations. The contribution from genuine two-particle correlations (signal) is weighted with $p_A$ (green line). For large *M* this contribution is suppressed when compared with two distinct contributions from a combinatorial background (red lines). The results from this figure cover any case in which particles are produced in pairs from the general two-variate p.d.f. $f_{xy}(x,y)$

The combinatorial weights $p_A$, $p_B$ and $p_C$ are universal—they depend only on multiplicity *M* and not on any details of the starting p.d.f. $f_{xy}(x,y)$. The universal multiplicity dependence of these two-particle combinatorial weights is presented in Fig. 2. We stress that the example analytic result in Eq. (16) can be further generalized by including the contributions when misidentified particles corresponding to the same initial particle type are coupled directly to each other in the two-particle correlators.

The practical use case of the above result amounts to the fact that we can now decompose and study separately the individual contributions to the measured two-particle correlators in the randomized sample and deduce their relative importance. For instance, the standard two-particle azimuthal correlation used in flow analyses can be calculated in the randomized sample as follows:

17 $$\begin{aligned} \langle 2\rangle\equiv & {} \langle \cos [n(\varphi _1\!-\!\varphi _2)]\rangle \nonumber \\ {}= & {} \int \!\!\!\!\int \cos [n(\varphi _1\!-\!\varphi _2)]w(\varphi _1,\varphi _2) \,\mathrm{{d}}\varphi _1 \mathrm{{d}}\varphi _2\,, \end{aligned}$$

where the p.d.f. *w*(*x*, *y*) is given by Eq. (16), while the azimuthal angles $\varphi _1$ and $\varphi _2$ can in general be taken from different sample spaces. The above equation describes analytically what is measured experimentally when we use two-particle azimuthal correlations, for the case when particles are emitted pair-wise, when the final dataset is randomized, and when only the most important cases of particle misidentification are taken into account. Given the nature of the example expression for the p.d.f. *w*(*x*, *y*) in Eq. (16), we are naturally led to decompose distinct individual contributions to the two-particle correlator as follows:

18 $$\begin{aligned} \langle 2\rangle = \langle 2\rangle _{w_1} + \langle 2\rangle _{w_2} + \langle 2\rangle _{w_3} + \langle 2\rangle _{w_4}\,, \end{aligned}$$

where

19 $$\begin{aligned} \langle 2\rangle _{w_1}\equiv & {} p_A \int \!\!\!\!\int \cos [n(\varphi _1\!-\!\varphi _2)] f_{\varphi _1\varphi _2}(\varphi _1,\varphi _2) \,\mathrm{{d}}\varphi _1 \mathrm{{d}}\varphi _2\,,\nonumber \\ \langle 2\rangle _{w_2}\equiv & {} p_B \int \!\!\!\!\int \cos [n(\varphi _1\!-\!\varphi _2)] f_{\varphi _1}(\varphi _1)f_{\varphi _2}(\varphi _2) \,\mathrm{{d}}\varphi _1 \mathrm{{d}}\varphi _2\,,\nonumber \\ \langle 2\rangle _{w_3}\equiv & {} p_C \int \!\!\!\!\int \cos [n(\varphi _1\!-\!\varphi _2)] f_{\varphi _1}(\varphi _1)f_{\varphi _1}(\varphi _2) \,\mathrm{{d}}\varphi _1 \mathrm{{d}}\varphi _2\,,\nonumber \\ \langle 2\rangle _{w_4}\equiv & {} p_C \int \!\!\!\!\int \cos [n(\varphi _1\!-\!\varphi _2)] f_{\varphi _2}(\varphi _1)f_{\varphi _2}(\varphi _2) \,\mathrm{{d}}\varphi _1 \mathrm{{d}}\varphi _2,\nonumber \\ \end{aligned}$$

and $f_{\varphi _1}(\varphi _1)\equiv \int f_{\varphi _1\varphi _2}(\varphi _1,\varphi _2)\,\mathrm{{d}}\varphi _2$ and analogously $f_{\varphi _2}(\varphi _2)\equiv \int f_{\varphi _1\varphi _2}(\varphi _1,\varphi _2)\,\mathrm{{d}}\varphi _1$. We illustrate and support this general decomposition with the toy Monte Carlo study in the next section.

#### Toy Monte Carlo study for the two-particle case

We test the validity of Eq. (16) with the following clear-cut toy Monte Carlo study, which can be solved analytically. The starting normalized two-variate p.d.f. is

20 $$\begin{aligned} f(\varphi _1,\varphi _2;M) = \frac{9375 \left( \varphi _1-\frac{M \varphi _2^2}{100}\right) ^2}{4 \pi ^4 \left( 3 \pi ^2 M^2-250 \pi M+12500\right) }\,, \end{aligned}$$

where *M* is a parameter and corresponds to multiplicity. The stochastic variables are azimuthal angles $\varphi _1$ and $\varphi _2$, whose sample space is $[0,2\pi )$. One can easily check that

21 $$\begin{aligned} \int _0^{2\pi }\!\!\!\!\int _0^{2\pi }f(\varphi _1,\varphi _2;M)\,\mathrm{{d}}\varphi _1 \mathrm{{d}}\varphi _2 = 1\,. \end{aligned}$$

for any value of multiplicity *M*.

**Fig. 3:**
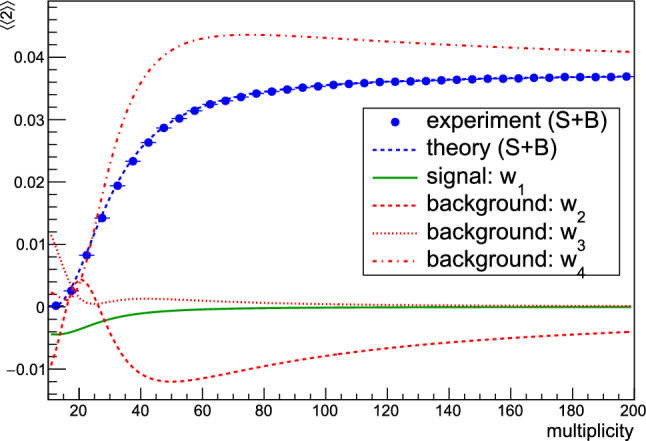
Analytic description of combinatorial background in two-particle correlations, when particles are produced in pairs, and the final data sample is randomized

With straightforward calculus, and after recalling that the combinatorial weight of the signal contribution for the two-particle case is $p_A = \frac{2!\frac{M}{2}}{M(M-1)}$, we have analytically obtained for flow harmonic $n=2$ for the signal contribution:

22 $$\begin{aligned} \langle 2\rangle _{w_1}= & {} p_A\int _0^{2\pi }\!\!\!\!\int _0^{2\pi }\cos [2(\varphi _1-\varphi _2)] f(\varphi _1,\varphi _2;M) \,\mathrm{{d}}\varphi _1 \mathrm{{d}}\varphi _2\nonumber \\= & {} -\frac{375 M}{4 \pi (M-1) (\pi M (3 \pi M-250)+12500)}\,, \end{aligned}$$

and similarly for the three distinct background contributions $\langle 2\rangle _{w_2}$, $\langle 2\rangle _{w_3}$ and $\langle 2\rangle _{w_4}$. All results are shown in Fig. 3. The blue markers show the experimental result, i.e. the average $\langle 2\rangle = \langle \cos [2(\varphi _1\!-\!\varphi _2)]\rangle$ measured in the randomized data sample, after particle azimuthal angles were sampled pair-wise from the p.d.f. in Eq. (20). The analytic expression for the signal contribution from Eq. (22) is shown with the green curve. Red curves represent analytic results for three distinct cases of combinatorial background contribution, $\langle 2\rangle _{w_2}$, $\langle 2\rangle _{w_3}$ and $\langle 2\rangle _{w_4}$, respectively. Finally, the blue curve is a superposition of the signal (green curve) and distinct background contributions (three red curves).

### Example solution for combinatorial background for three-particle correlations

In this section, we address the combinatorial background for three-particle correlations. The problem we try to solve can be mathematically formulated as follows:

1. From the general three-variate p.d.f. $f_{xyz}(x,y,z)$ particles are emitted in triples. We perform *M*/3 samplings to obtain an event with multiplicity *M*.
2. We randomize the final sample of *M* particles.
3. What is the p.d.f. *w*(*x*, *y*, *z*) of the randomized sample, if the starting p.d.f. is $f_{xyz}(x,y,z)$?

Physically, $f_{xyz}(x,y,z)$ describes the “signal,” while *w*(*x*, *y*, *z*) describes the “signal + background.”

For instance, if we are interested in the average three-particle correlation $\langle xyz\rangle$, then the signal contribution is given by $\langle xyz\rangle _{S}=\int \!\!\int \!\!\int \! xyz\, f_{xyz}(x,y,z)\, \mathrm{{d}}x\mathrm{{d}}y\mathrm{{d}}z$, while the contribution from both signal and background is given by $\langle xyz\rangle _{S+B}=\int \!\!\int \!\!\int \! xyz\, w(x,y,z)\, \mathrm{{d}}x\mathrm{{d}}y\mathrm{{d}}z$. Given that we have only randomized the initial sample, there should be no systematic biases involved, i.e. we seek the universal relation between *w*(*x*, *y*, *z*) and $f_{xyz}(x,y,z)$. The derivation of that universal relation is the main result of this section—its generic structure deciphers the role of combinatorial background after we have randomized the final data sample for any starting p.d.f. $f_{xyz}(x,y,z)$.

We keep the discussion both general and as close to the experiment as possible. For instance, we introduce particle misidentification by blinding the particle labels *x*, *y*, *z* in the final data sample. This corresponds to the real-case scenario when, for instance, three pions are emitted from three different physical processes. After randomization, we cannot deduce even in principle which pion originated from which process, since identical particles are indistinguishable. Just as in the two-particle example, we consider here only this most important case of particle misidentification. The more general solutions which cover all possible cases of particle misidentification will be presented in our future work.

If particles are sampled in triples from $f_{xyz}(x,y,z)$, and if we consider only the most important case of particle misidentifications, without loss of generality we can always divide the sample space after randomization into the following three disjoint categories:

1. **Diagonal:** All three particles are from the same sampling. This case is governed by $f_{xyz}$, i.e. this is the signal. The combinatorial weight of this contribution is $p_{A} = \frac{3!\frac{M}{3}}{M(M-1)(M-2)}$.
2. **Semi-diagonal:** Only two particles are from the same sampling. In order to write down p.d.f.’s which govern this case, we have to further differentiate into two disjoint subcategories with different statistical properties. Their corresponding p.d.f.’s and combinatorial weights are

- $f_{xy}f_{x}$, $f_{xy}f_{y}$, $f_{xz}f_{x}$, $f_{xz}f_{z}$, $f_{yz}f_{y}$ and $f_{yz}f_{z}$. These cases correspond to the situation when two particles of different types originate from the same sampling. To account for the possibility that there is a genuine two-particle correlation between them, we use marginal two-particle p.d.f.’s. The third particle is of the same type, but it originated from separate sampling. In total, there are six different possibilities, and the combinatorial weight of each is $p_{B_{1}} = \frac{3!\frac{M}{3}(\frac{M}{3}-1)}{M(M-1)(M-2)}$;
- $f_{xy}f_{z}$, $f_{xz}f_{y}$, and $f_{yz}f_{x}$. This is similar to the previous case; the only difference is that the third particle is of a different type. There are three different possibilities, and the combinatorial weight of each is $p_{B_{2}} = \frac{3!\frac{M}{3}(\frac{M}{3}-1)}{M(M-1)(M-2)}$.

3. **Off-diagonal:** All three particles are from different samplings. To write down the universal decomposition due to the combinatorial background in this sector, we must further decompose into three disjoint subcategories:

- $f_{x}f_{x}f_{x}$, $f_{y}f_{y}f_{y}$, $f_{z}f_{z}f_{z}$. This is the case when in the three-particle correlation we have three particles of the same type, each of which originated from separate sampling. There are three distinct cases, and the combinatorial weight of each is $p_{C_{1}} = \frac{\frac{M}{3}(\frac{M}{3}-1)(\frac{M}{3}-2)}{M(M-1)(M-2)}$;
- $f_{x}f_{x}f_{y}$, $f_{x}f_{x}f_{z}$, $f_{y}f_{y}f_{x}$, $f_{y}f_{y}f_{z}$, $f_{z}f_{z}f_{x}$, $f_{z}f_{z}f_{y}$. In this subcategory, we have three particles from three different samplings, two of which are from the same type. There are six distinct possibilities, and the combinatorial weight of each is $$p_{C_{2}} = \frac{\left( {\begin{array}{c}3\\ 2\end{array}}\right) \frac{M}{3}(\frac{M}{3}-1)(\frac{M}{3}-2)}{M(M-1)(M-2)}$$;
- $f_{x}f_{y}f_{z}$. Finally, this case corresponds to the situation when we have three different particles from three different samplings in the three-particle correlator. The combinatorial weight is $p_{C_{3}} = \frac{3!\frac{M}{3}(\frac{M}{3}-1)(\frac{M}{3}-2)}{M(M-1)(M-2)}$.

In total, in this example there are 20 disjoint cases to deal with: one from the signal and 19 from the combinatorial background. We can check immediately that the corresponding probabilities sum up correctly:

23 $$\begin{aligned} \sum _i^{20} p_i= & {} p_{A} + 6p_{B_{1}} + 3p_{B_{2}} + 3p_{C_{1}} + 6p_{C_{2}} + p_{C_{3}}\nonumber \\= & {} \frac{2M + 9\cdot 6\frac{M}{3}\left( \frac{M}{3}-1\right) + \left( 3+6\left( {\begin{array}{c}3\\ 2\end{array}}\right) +3!\right) \frac{M}{3}\left( \frac{M}{3}-1\right) \left( \frac{M}{3}-2\right) }{M(M-1)(M-2)} \nonumber \\ {}= & {} 1\,. \end{aligned}$$

**Fig. 4:**
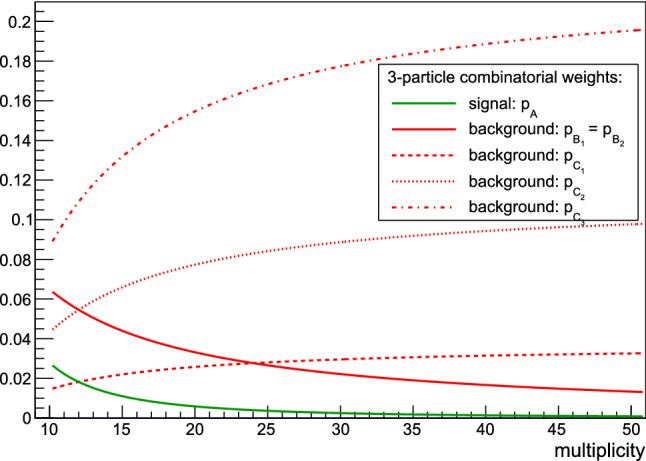
Multiplicity dependence of six combinatorial weights for three-particle correlations: $p_A$, $p_{B_1}$, $p_{B_2}$, $p_{C_1}$, $p_{C_2}$ and $p_{C_3}$ (see the main text for their definitions and further explanation). The contribution from genuine three-particle correlations (signal) is weighted with $p_A$ (green line), and already for $M\approx 50$ this contribution becomes negligible. The contribution from genuine two-particle correlations is also suppressed for large multiplicities (solid red line). The remaining weights, $p_{C_1}$, $p_{C_2}$ and $p_{C_3}$, all correspond to independent particle emission, and their relative contribution increases as multiplicity increases

From the above decomposition we can now read off and write down our final solution for this particular example. If the starting p.d.f. is $f_{xyz}(x,y,z)$, the p.d.f. *w*(*x*, *y*, *z*) after randomization is given by the following universal result:

24 $$\begin{aligned} w(x,y,z)= & {} p_A f_{xyz}(x,y,z)\nonumber \\&+ p_{B_1}\big [f_{xy}(x,y)f_{x}(z) + f_{xy}(x,y)f_{y}(z) + f_{xz}(x,z)f_{x}(y)\nonumber \\&+ f_{xz}(x,z)f_{z}(y) + f_{yz}(y,z)f_{y}(x) + f_{yz}(y,z)f_{z}(x)\big ]\nonumber \\&+ p_{B_2}\big [f_{xy}(x,y)f_{z}(z) + f_{xz}(x,z)f_{y}(y) + f_{yz}(y,z)f_{x}(x)\big ]\nonumber \\&+ p_{C_1}\big [f_{x}(x)f_{x}(y)f_{x}(z) + f_{y}(x)f_{y}(y)f_{y}(z) \nonumber \\&+ f_{z}(x)f_{z}(y)f_{z}(z)\big ]+ p_{C_2}\big [f_{x}(x)f_{x}(z)f_{y}(y)\nonumber \\&+ f_{x}(x)f_{x}(y)f_{z}(z) + f_{y}(y)f_{y}(z)f_{x}(x)\nonumber \\&+ f_{y}(y)f_{y}(x)f_{z}(z) \nonumber \\&+ f_{z}(z)f_{z}(y)f_{x}(x) + f_{z}(z)f_{z}(x)f_{y}(y) \big ]\nonumber \\&+ p_{C_3}f_{x}(x)f_{y}(y)f_{z}(z)\,. \end{aligned}$$

This result is universal because the six combinatorial weights $p_A$, $p_{B_1}$, $p_{B_2}$, $p_{C_1}$, $p_{C_2}$ and $p_{C_3}$ depend only on multiplicity, while all marginal p.d.f.’s are always by definition calculated in the same way from the starting p.d.f. $f_{xyz}(x,y,z)$ (for instance, $f_{xy}(x,y) \equiv \int f_{xyz}(x,y,z)\,\mathrm{{d}}z$, $f_{x}(x) \equiv \int \!\!\int f_{xyz}(x,y,z)\,\mathrm{{d}}y\mathrm{{d}}z$, etc.). Therefore, whatever the starting p.d.f. $f_{xyz}(x,y,z)$ is, one needs to calculate all marginal p.d.f.’s and insert them in the above equation, and the result for the p.d.f. *w*(*x*, *y*, *z*) of the randomized sample follows. We stress again that the example analytic result in Eq. (24) can be further generalized by including the contributions when misidentified particles corresponding to the same initial particle type are coupled directly to each other in the three-particle correlators.

Figure 4 shows the multiplicity dependence of six combinatorial weights for three-particle correlations: $p_A$, $p_{B_1}$, $p_{B_2}$, $p_{C_1}$, $p_{C_2}$ and $p_{C_3}$. As expected, all weights that correspond either to genuine three-particle correlation ($p_A$) or genuine two-particle correlations ($p_{B_{1}}$ and $p_{B_{2}}$) diminish rather quickly as multiplicity increases.

In the next section, we use the clear-cut toy Monte Carlo study to confirm the validity of our main result in Eq. (24).

#### Toy Monte Carlo study for the three-particle case

In this section, we set up a toy Monte Carlo study to confirm the validity of our main result in Eq. (24). We organize the study in such a way that it does not lead to simplifications at any step. For instance, we choose random observables *x*, *y* and *z* to have different functional forms of single-variate marginal p.d.f.’s, defined over different sample spaces.

The starting three-variate p.d.f. *f*(*x*, *y*, *z*; *M*) is given by the following expression:

25 $$\begin{aligned} f(x,y,z;M)\equiv & {} \frac{100+Mx+2My+3Mz}{6(100+7M)},\nonumber \\&x\in (0,1),y\in (0,2),z\in (0,3)\,. \end{aligned}$$

As defined above, the three stochastic variables are *x*, *y* and *z*, while *M* is a parameter that corresponds to multiplicity. The normalization constraint is satisfied for any *M*, i.e.

26 $$\begin{aligned} \int _0^1\!\!\!\int _0^2\!\!\!\int _0^3 f(x,y,z;M) \,\mathrm{{d}}x\mathrm{{d}}y\mathrm{{d}}z= & {} 1\,,\forall M\,. \end{aligned}$$

This toy model was carefully designed with the following two key aspects in mind: (a) it can be solved analytically; (b) all individual terms in Eq. (24) have different non-vanishing contributions (i.e. there are no underlying symmetries due to which some terms would be identically zero). From the p.d.f. in Eq. (25), we sample three particles *M*/3 times to obtain an event with multiplicity *M*. The true value of the average three-particle correlation (signal), $\langle xyz\rangle _S$, can be obtained with the straightforward calculus. We have obtained

27 $$\begin{aligned} \langle xyz\rangle _S= & {} \int _0^1\!\!\!\int _0^2\!\!\!\int _0^3 xyz f(x,y,z;M) \,\mathrm{{d}}x\mathrm{{d}}y\mathrm{{d}}z\nonumber \\= & {} \frac{75+7M}{100+7M}\,. \end{aligned}$$

Now we proceed with the non-trivial part. In each event we randomize the sampled variables, and calculate the average three-particle correlation $\langle xyz\rangle _{S+B}$ in the randomized sample (signal + background). To get the theoretical result for $\langle xyz\rangle _{S+B}$, in the first step we calculate all marginal p.d.f.’s $f_{xy},f_{xz},f_{yz},f_{x},f_{y}$ and $f_{z}$ from the starting three-variate p.d.f. *f*(*x*, *y*, *z*; *M*) in Eq. (25). For instance, for the two-variate marginal p.d.f. of *x* and *y* we have obtained the following analytic result:

28 $$\begin{aligned} f_{xy}(x,y;M) = \frac{200+M(9+2x+4y)}{400+28M}\,. \end{aligned}$$

We remark that by definition, if the starting p.d.f. is normalized, any marginal p.d.f.’s calculated from it are automatically normalized; for example, we have

29 $$\begin{aligned} \int _0^1\!\!\!\int _0^2 f_{xy}(x,y;M)\,\mathrm{{d}}x\mathrm{{d}}y = 1\,,\forall M\,. \end{aligned}$$

After we have obtained all marginal p.d.f.’s $f_{xy},f_{xz},f_{yz},f_{x},f_{y}$ and $f_{z}$, we insert them in Eq. (24), together with universal combinatorial weights for the three-particle case $p_A$, $p_{B_1}$, $p_{B_2}$, $p_{C_1}$, $p_{C_2}$, and $p_{C_3}$, to obtain the analytic result for the p.d.f. of the randomized sample *w*(*x*, *y*, *z*). Finally, with respect to this p.d.f., we calculate the average three-particle correlation, which now analytically describes the contributions from both the signal and the combinatorial background. After some straightforward calculus, we have obtained our final result:

30 $$\begin{aligned} \langle xyz\rangle _{S+B}= & {} \int _0^1\!\!\!\int _0^2\!\!\!\int _0^3 xyz\, w(x,y,z;M) \,\mathrm{{d}}x\mathrm{{d}}y\mathrm{{d}}z\nonumber \\= & {} \frac{9000000+2M(-4132500+M(316475+M(258423+4M(6487+192M))))}{3(M\!-\!1)(M\!-\!2)(7M\!+\!100)^3}\,. \end{aligned}$$

**Fig. 5:**
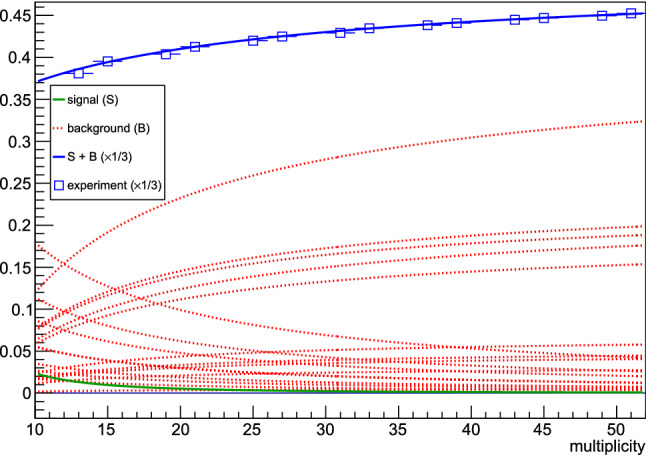
Example analytic description of combinatorial background in three-particle correlations, when particles are produced in triples, and the final data sample is randomized

These results are shown in Fig. 5. Blue markers show the experimental result, i.e. the average $\langle xyz\rangle$ measured in the randomized data sample. Both signal and combinatorial background contribute to this measurement. The analytic expression for the signal contribution is shown with the green curve—this is the result from Eq. (27) weighted with its combinatorial weight $p_{A} = \frac{3!\frac{M}{3}}{M(M-1)(M-2)}$. Red curves represent analytic results for 19 distinct cases of combinatorial background contribution, weighted with corresponding combinatorial weights $p_{B_{1}}, p_{B_{2}}, p_{C_{1}}, p_{C_{2}}$ and $p_{C_{3}}$ (see Eq. (24)). Finally, the blue curve is given by Eq. (30), and it is the sum of the signal (green curve) and distinct background contributions (19 red curves). This study demonstrates that the example theoretical result from Eq. (24) describes with great precision the measurement for the three-particle correlation in the randomized data sample when both signal and combinatorial background contributions are present.

The result in Eq. (24) can be further generalized and fine-tuned by adding the terms corresponding to subdominant cases of particle misidentification, but conceptually the structure of the solution is always the same. The relation between the original p.d.f. *f* and its counterpart *w* in the randomized dataset is universal, in the sense that the relation is determined solely by combinatorial weights $p_i$, which depend only on multiplicity, and marginal p.d.f.’s of *f*.

## Summary

In summary, we have discussed the role of reflection symmetry, permutation symmetry, frame independence, and relabeling of particle indices in the cumulant expansion. The conclusions are general and applicable to cumulants of any stochastic variables; however, the primary aim was to further clarify the properties of the recently proposed event-by-event cumulants of azimuthal angles. For the first time, the analytic solutions for the contribution of combinatorial background in the measured two- and three-particle correlations were derived. The main result is the demonstration that these solutions for the combinatorial background are universal—they can be written generically in terms of multiplicity-dependent combinatorial weights and marginal probability density functions of starting multivariate distribution. Finally, the new general results between expectation values of multiparticle azimuthal correlators and flow amplitudes and symmetry planes were presented.

## Data Availability

This manuscript has no associated data or the data will not be deposited. [Authors’ comment: The manuscript has no associated data.]

## Appendix A: Expectation values

In this appendix, we summarize the theoretical expectation values of all estimators used in this work. Since our estimators are defined in terms of sums, where each summand has the same generic expression, their expectation values are trivially related to the expectation values of summands, i.e. the fundamental building blocks in the sums. Therefore, we first derive the theoretical expectation values of summands, which we denote as fundamental observables, and from them trivially deduce the expectation values of high-level compound observables. In all derivations we assume that only anisotropic flow correlations are present; therefore, for an event with multiplicity *M* we can use $f(\varphi _1,\varphi _2,\ldots ,\varphi _M) = f(\varphi _1)f(\varphi _2)\cdots f(\varphi _M)$, where every single-particle p.d.f. on the right-hand side is parameterized with the same normalized Fourier series (see introductory part of Ref. [11] for further details).

### Fundamental observables

For the sake of completeness, and to unify the notation, we first reproduce the theoretical expectation value of the *k*-particle azimuthal correlator evaluated in harmonics $n_1, \ldots , n_k$, in a data sample containing *M* azimuthal angles ($k\le M$):

A.1 $$\begin{aligned}&E[e^{i(n_1\varphi _1+\cdots +n_k\varphi _k)}] \equiv \left<e^{i(n_1\varphi _1+\cdots +n_k\varphi _k)}\right>\nonumber \\= & {} \int \cdots \int e^{i(n_1\varphi _1+\cdots +n_k\varphi _k)}f(\varphi _1,\cdots ,\varphi _M)\,\mathrm{{d}}\varphi _1 \ldots \mathrm{{d}}\varphi _M\nonumber \\= & {} \int \cdots \int e^{in_1\varphi _1}\cdots e^{in_k\varphi _k}f(\varphi _1)\cdots f(\varphi _M)\,\mathrm{{d}}\varphi _1 \ldots \mathrm{{d}}\varphi _M\nonumber \\= & {} \left( \prod _{i=1}^{k}\int e^{in_i\varphi _i}f(\varphi _i)\,\mathrm{{d}}\varphi _i\right) \times \left( \prod _{i=k+1}^{M}\int f(\varphi _i)\,\mathrm{{d}}\varphi _i\right) \nonumber \\= & {} \left( \prod _{i=1}^{k} v_{n_i}e^{in_i\varPsi _{n_i}}\right) \times 1\nonumber \\= & {} v_{n_1}\cdots v_{n_k}e^{i(n_1\varPsi _{n_1}+\cdots +n_k\varPsi _{n_k})}\,. \end{aligned}$$

This very important analytic result was first derived in Ref. [17]. From the above general result we can read off directly the following specific results:

A.2 $$\begin{aligned} E[\cos n\varphi _i]= & {} v_n\cos \varPsi _n\,,\quad \forall n,i \,,\end{aligned}$$

A.3 $$\begin{aligned} E[\sin n\varphi _i]= & {} v_n\sin \varPsi _n\,,\quad \forall n,i \,,\end{aligned}$$

A.4 $$\begin{aligned} E[\cos [n(\varphi _i-\varphi _j)]]= & {} v_n^2\,,\quad \forall n,i,j\ (i\ne j) \,,\end{aligned}$$

A.5 $$\begin{aligned} E[\sin [n(\varphi _i-\varphi _j)]]= & {} 0 \,,\quad \forall n,i,j\ (i\ne j) \,. \end{aligned}$$

By following a similar procedure, we have derived the following new general results for additional expectation values of interest:

A.6 $$\begin{aligned} E[\cos ^{2p}n\varphi _i]= & {} \frac{1}{2^{2p-1}}\left[ \frac{1}{2}\left( {\begin{array}{c}2p\\ p\end{array}}\right) + \sum _{k=0}^{p-1}\left( {\begin{array}{c}2p\\ k\end{array}}\right) v_{2(p-k)n}\right. \nonumber \\&\left. \cos [2(p-k)n\varPsi _{2(p-k)n}] \right] \,,\nonumber \\ E[\cos ^{2p-1}n\varphi _i]= & {} \frac{1}{2^{2p-2}}\sum _{k=0}^{p-1}\left( {\begin{array}{c}2p-1\\ k\end{array}}\right) v_{(2p-2k-1)n}\nonumber \\&\cos [(2p-2k-1)n\varPsi _{(2p-2k-1)n}]\,,\nonumber \\ E[\sin ^{2p}n\varphi _i]= & {} \frac{1}{2^{2p-1}}\left[ \frac{1}{2}\left( {\begin{array}{c}2p\\ p\end{array}}\right) + \sum _{k=0}^{p-1}(-1)^{p-k}\right. \nonumber \\&\left. \left( {\begin{array}{c}2p\\ k\end{array}}\right) v_{2(p-k)n} \cos [2(p-k)n\varPsi _{2(p-k)n}] \right] \,,\nonumber \\ E[\sin ^{2p-1}n\varphi _i]= & {} \frac{1}{2^{2p-2}}\sum _{k=0}^{p-1}(-1)^{p+k-1}\nonumber \\&\left( {\begin{array}{c}2p-1\\ k\end{array}}\right) v_{(2p-2k-1)n}\nonumber \\&\sin [(2p-2k-1)n\varPsi _{(2p-2k-1)n}]\,. \end{aligned}$$

In the derivation, we have used result 1.320 from Ref. [18] in combination with the orthogonality relations of trigonometric functions. As an example, we can now easily read off the following specific cases:

A.7 $$\begin{aligned} E[\cos ^2 n\varphi _i]= & {} \frac{1}{2}\left[ 1 + v_{2n}\cos (2n\varPsi _{2n})\right] \,,\quad \forall n,i\,,\end{aligned}$$

A.8 $$\begin{aligned} E[\cos ^3 n\varphi _i]= & {} \frac{1}{4}\left[ 3v_{n}\cos (n\varPsi _{n}) + v_{3n}\cos (3n\varPsi _{3n})\right] ,\,\nonumber \\&\quad \quad \forall n,i\,,\end{aligned}$$

A.9 $$\begin{aligned} E[\sin ^2 n\varphi _i]= & {} \frac{1}{2}\left[ 1 - v_{2n}\sin (2n\varPsi _{2n})\right] \,,\quad \forall n,i\,,\end{aligned}$$

A.10 $$\begin{aligned} E[\sin ^3 n\varphi _i]= & {} \frac{1}{4}\left[ 3v_{n}\cos (n\varPsi _{n}) - v_{3n}\sin (3n\varPsi _{3n})\right] ,\,\nonumber \\&\quad \quad \forall n,i\,. \end{aligned}$$

In fact, the results in Eq. (A.6) suffice to decipher the expectation values of most general products, because

A.11 $$\begin{aligned}&E\bigg [\bigg (\prod _{i=1}^{k_c}\cos ^{p_i}n_i\varphi _i\bigg )\bigg (\prod _{j=k_c+1}^{k_s}\sin ^{r_j}m_j\varphi _j\bigg )\bigg ]\nonumber \\&\quad = \prod _{i=1}^{k_c}E[\cos ^{p_i}n_i\varphi _i] \prod _{j=k_c+1}^{k_s}E[\sin ^{r_j}m_j\varphi _j]\,. \end{aligned}$$

For instance

A.12 $$\begin{aligned} E[\cos n_1\varphi _i\cos n_2\varphi _j]= & {} v_{n_1}v_{n_2}\cos \varPsi _{n_1}\cos \varPsi _{n_2},\,\nonumber \\&\quad \quad \forall n_1,n_2,i,j\ (i\ne j)\,,\end{aligned}$$

A.13 $$\begin{aligned} E[\sin n_1\varphi _i\sin n_2\varphi _j]= & {} v_{n_1}v_{n_2}\sin \varPsi _{n_1}\sin \varPsi _{n_2},\,\nonumber \\&\quad \quad \forall n_1,n_2,i,j\ (i\ne j)\,, \end{aligned}$$

We now demonstrate that the expectation values of all high-level observables can be reduced to these fundamental ones.

### Compound observables

The expectation value of any compound observable can now be trivially deduced from the fundamental expectation values presented in the previous section. For instance, for the single-event average two-particle correlation, $\langle 2\rangle$, we use the following statistics built from azimuthal angles $\varphi _1, \ldots , \varphi _M$ (*M* is the multiplicity of an event

A.14 $$\begin{aligned} \langle 2\rangle \equiv \frac{1}{M(M-1)}\sum _{i\ne j}^M \cos (\varphi _i-\varphi _j)\,. \end{aligned}$$

Its expectation value is

A.15 $$\begin{aligned} E[\langle 2\rangle ]= & {} \frac{1}{M(M-1)}\sum _{i\ne j}^M E[\cos (\varphi _i-\varphi _j)]\nonumber \\= & {} \frac{1}{M(M-1)}\sum _{i\ne j}^M v^2\nonumber \\= & {} \frac{1}{M(M-1)} M(M-1) v^2\nonumber \\= & {} v^2\,. \end{aligned}$$
